# The True Law of Population

**Published:** 1873-06

**Authors:** Nathan Allen

**Affiliations:** Lowell, Mass.


The following article is copied from the *Medical Press and Circular*, of Feb. 5th, 1873, which is published simultaneously in London, Edinburgh and Dublin, and is one of the oldest and most extensively circulated medical journals in Great Britain. The article was prepared expressly for that periodical by the request of its editor, Professor James, and contains, as it states, only a synopsis of Dr. Allen's views on the subject.

### *The True Law of Population. Based on Physiology and Psychology.*

By Dr. NATHAN ALLEN, of Lowell, Mass.

It may appear almost presumptuous to assume the phrase, "true law," which might seem to imply that all other theories were false or not true; but such is not our meaning. It is intended to express this sentiment, that whatever views are entertained on this subject, or however diverse they are, they must all, as far as true, be subservient to a great general law which has its origin and foundation in physiology.

More than thirty years ago, a work was published in London with this title, "The True Law of Population shown to be connected with the Food of the People." The merits of this work consisted very much in the evidences which physiology afforded in support of its theory, arising from the effects that food combined with other agencies had in changing physical organization. But, unfortunately, Mr. Doubleday, the author of this work, as well as some other writers upon population, have not been thoroughly educated in the principles of this science.

As this subject is so vast and complicated, a large volume would be required to discuss it properly, especially in expounding any new views; we can present here only a few points or heads of topics, by way of suggestion and illustration. An examination into the views and theories of most writers upon population shows that the laws which they lay down for its increase have been controlled generally by agents or objects entirely external to the body, and some of them hold only remote or indirect relations to it. Now, while these external agents, such as food, climate, exercise, etc., may operate as powerful factors or as secondary causes, we maintain that there is a great general law of propagation which extends through the whole animal and vegetable kingdoms. Whatever influences these agencies may have in the development of the body, the most important agent or law of all, the law that shapes its life, character, and destiny, it would seem, must have its origin and seat somewhere in the body itself. What then is this law? It may be defined thus: *It consists in the perfectionism*

*of structure and harmony of function;* or, in other words, that every organ of the body should be perfect in its structure, and that each should perform its legitimate functions in harmony with all others.

While this perfect physical organization is nowhere to be found in nature, we can readily conceive of such a standard, and that there may be all manner of approximations towards it. The nearer this standard is reached, the more completely the law of propagation can be carried out. This theory is supported by evidences deduced from physiology in a variety of ways.

All diseases interfere with the operation of this law, especially those that are considered hereditary. This class of diseases become intensified by each generation, and tend rapidly not only to impair the vitality, but to blot out the existence of the race. There is another class of diseases or weaknesses, described under "sterility," "barrenness," and "impotence," from which strong evidences may be derived.

There is a general principle in physiology, favorable to this theory, which is thus described by Dr. Carpenter:

"There is a certain antagonism between the nutritive and reproductive functions, the one being exercised at the expense of the other. The reproductive apparatus derives the materials of its operations through the nutritive system and its functions. If, therefore, it is in a state of excessive activity, it will necessarily draw off from the individual fabric some portion of aliment destined for its maintenance. It may be universally observed that when the nutritive functions are particularly active in supporting the individual, the reproductive system is undeveloped, and *vice versa.*" Let, therefore, any class of organs or any parts of the body be unduly or excessively exercised, and it requires the more nutrition to support them, thereby withdrawing what should go to other organs. In accordance with this physiological law, if any class of organs become predominant in their development, if what may properly be denominated one of the temperaments becomes excessively developed, it conflicts with this great law of increase. In other words, if the organization is carried by successive generations to an extreme—that is, to a high nervous temperament—a predominance of the brain and nervous system; or, on the other hand, to a lymphatic temperament—a predominance of the mere animal nature, it operates unfavorably upon the increase of progeny. Accordingly in the highest states of refinement, culture and civilization of a people, the tendency has always been to run out in offspring; while on the other hand all tribes or races sunk in the lowest stages of barbarism, controlled principally by their animal nature, do not abound in offspring, and, in the course of time, they tend also to run out.

The same general fact has been observed among all the abnormal classes, such as idiots, cretins, the insane, the deaf and dumb, and, to some extent, with extreme or abnormal organizations, such as are excessively corpulent, or spare, as well as of unnatural size, either very large, or diminutively small.

A similar fact has been observed among distinct classes, such as the nobility, the aristocracy, etc., where by inheritance, refinement and culture the nervous temperament becomes very predominant; it is found that such families in the course of a few generations do not increase in offspring, and, not unfrequently, in time, they become extinct.

A similar result has also followed the intermarriage of relations, from the fact, that the same weaknesses or predispositions are intensified by this relation. Again, if we take those families and races which, for successive generations, have steadily increased most, we shall find that, as a whole, they possess a remarkably healthy, well-balanced organization. Illustrations of this type, we shall find, abound most among the middling or working classes of the Germans, the English, the Scotch, the Irish, and the Americans.

The laws of hereditary descent afford strong evidence in favor of some general law of propagation. The fact, that "like begets like," subject to certain variations and conditions, is a proverb that has been too well attested to be called in question. The union of two agents, possessing similar and dissimilar qualities, constitutes an important condition to which this law of propagation is subject. While it may be difficult to point out, in all cases, the exact results of hereditary influences, still it has been found on a large scale that, in the aggregate, there was the most unquestioned evidence of such agency, and that it was minute and extensive, and continued for successive generations. When this department of physiology becomes thoroughly understood, hereditary influences will more readily be traced back to their primary sources, and to the secondary causes operating to change and modify them. Now, the same evidence that proves the existence of hereditary agency, implies that there is somewhere a general law, of which this is part and parcel; and no one thing will throw so much light upon this whole subject of inheritance, as the recognition of a law of propagation, being based upon a perfect standard in nature. Again, there must exist in physiology a certain standard or type of organization for woman, best adapted for increase both for herself and her offspring. This, we believe, consists in a perfectly healthy and well-balanced body, that, with such an organization, the pregnant state harmonizes, the process of delivery is the safest, and the materials for nursing offspring are found most complete.

This theory of human increase derives evidence from an analogous law in the animal and vegetable kingdoms. It is well known that wonderful improvements have been made, within the present century, in domestic animals, and this chiefly, too, by an application of physiological laws. To such an extent have the results of observation and experiment been here carried that this process of improvement has been reduced almost to a science. The terms here used, "Pure Blood," "Thorough-Bred," "Pedigree," "Breeding in-and-in," and "Cross-Breeding," may all be explained by two great leading principles. One is, a general law of propagation based upon a perfect standard, and the other is, the law of inheritance subject to certain conditions.

In fact, these two laws constitute, we believe, the two great principles that underlie most of the theories of Charles Darwin; these are discussed under the head of "Natural Selection," and the "Law of Variability;" and whatever may be said against his theories of the "Origin of Species," and "Descent of Man," these two general principles are in our opinion founded in nature and will survive all opposition and criticism.

A similar law of propagation exists in vegetable physiology. It is a fact well attested by gardeners, that in order to produce flowers and fruit, the soil must not be too rich or too poor; if the plant or tree grows too luxuriously, its branches or roots must be pruned; while on the other hand, if unthrifty, it must receive better culture and its roots be enriched before it will become fruitful. So the most beautiful flowers and richest fruit have few seeds, which in time run out, while that of a poor quality may abound in seed, but will not flourish long. It is true the conditions here vary, and so do the modes of perpetuating life; but, by analogy, facts and arguments of a positive character may be gleaned from this source to confirm a similar law in human physiology.

Other facts and arguments might be adduced in favor of such a theory of population, did time and space permit, but we close with two or three suggestions. If this law of propagation is true, it presents a great standard for improvement, where there may be found the perfect free agency of man, and, at the same time, the highest motives for improvement. It could not have been intended by the Creator that man should always be ignorant of, or ignore, the great law of propagation, in many respects the most important in the universe; neither could it have been intended that man as a moral and accountable being should be a mere *passive agent* in the propagation of the species.

Again, this law of population should interest medical men, as it affords a new stand-point in physiology to study the relations of the body as a whole and especially in their connection with the reproductive organs, which have constituted the chief study on this subject. It presents also, in some respects, a new guide to female organization as to what is the normal or best standard for the propagation of the species, and it may throw light on the changes occasioned by pregnancy, as well as on the qualities indispensable for good nursing. It explains in a clearer light than ever not only the laws of hereditary descent, but magnifies their value and importance in relation to human progress and welfare. It opens new views connected with anthropology, and aids in explaining many changes in the history of different races and nations. Inasmuch as it furnishes a new key to the study of the body, and exalts physical laws in their connection with human improvement, it should certainly interest medical men.

From more than thirty years of observation and experience in a busy professional life we have become every year convinced more and more of the truth and importance of the law here so briefly discussed, and would commend it to the attention of all inquirers after truth in this great field of observation and study.

